# Roles of Lysine Methylation in Glucose and Lipid Metabolism: Functions, Regulatory Mechanisms, and Therapeutic Implications

**DOI:** 10.3390/biom14070862

**Published:** 2024-07-19

**Authors:** Zhen Wang, Huadong Liu

**Affiliations:** School of Health and Life Sciences, University of Health and Rehabilitation Sciences, Qingdao 266113, China; wangzhen@uor.edu.cn

**Keywords:** lysine methylation, glucose metabolism, lipid metabolism

## Abstract

Glucose and lipid metabolism are essential energy sources for the body. Dysregulation in these metabolic pathways is a significant risk factor for numerous acute and chronic diseases, including type 2 diabetes (T2DM), Alzheimer’s disease (AD), obesity, and cancer. Post-translational modifications (PTMs), which regulate protein structure, localization, function, and activity, play a crucial role in managing cellular glucose and lipid metabolism. Among these PTMs, lysine methylation stands out as a key dynamic modification vital for the epigenetic regulation of gene transcription. Emerging evidence indicates that lysine methylation significantly impacts glucose and lipid metabolism by modifying key enzymes and proteins. This review summarizes the current understanding of lysine methylation’s role and regulatory mechanisms in glucose and lipid metabolism. We highlight the involvement of methyltransferases (KMTs) and demethylases (KDMs) in generating abnormal methylation signals affecting these metabolic pathways. Additionally, we discuss the chemical biology and pharmacology of KMT and KDM inhibitors and targeted protein degraders, emphasizing their clinical implications for diseases such as diabetes, obesity, neurodegenerative disorders, and cancers. This review suggests that targeting lysine methylation in glucose and lipid metabolism could be an ideal therapeutic strategy for treating these diseases.

## 1. Introduction

Glucose and lipid metabolism are essential energy sources for organisms to sustain life activities [1]. An imbalance in glucose and lipid metabolism is a high-risk factor leading to various complications, including obesity, diabetes, hyperlipidemia, nonalcoholic fatty liver disease (NAFLD), and tumors [2]. A variety of proteins, including key enzymes, regulate these metabolic processes [3]. Post-translational modifications (PTMs) are among the most critical factors affecting the activity and function of these proteins [4]. PTMs involve enzymatic or chemical processes that introduce covalent groups into the side chains or terminals of amino acids in proteins [5]. These modifications alter the physicochemical properties of proteins involved in glucose and lipid metabolism, impacting protein structure, localization, activity, and binding partners [6]. Recent evidence indicates that PTMs play a crucial role in diseases resulting from aberrant glucose and lipid metabolism [7]. In the past few decades, lysine methylation has been established as a significant PTM in the human proteome [8]. Emerging as a biologically meaningful PTM, lysine methylation has recently gained attention for its role in human diseases [9]. This review examines lysine methylation and its role in glucose and lipid metabolism.

**Figure 1:**
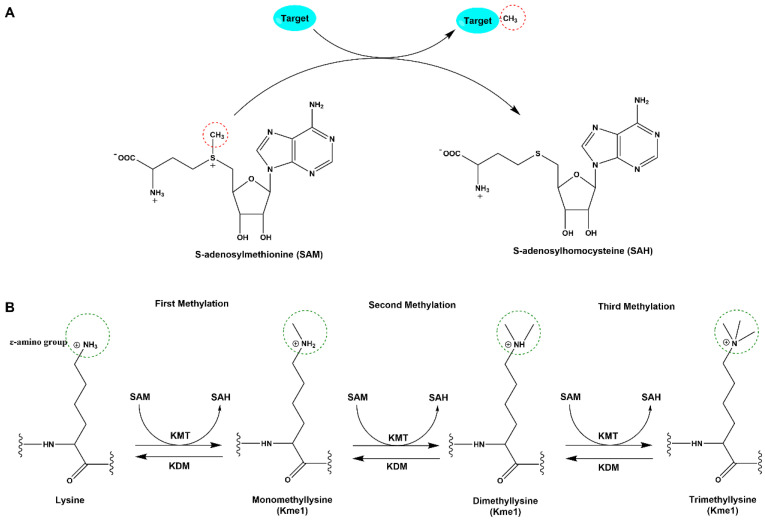
Schematic diagram of the methyltransferase catalytic reaction. (**A**) In each lysine methylation reaction, the methyl donor cofactor SAM is converted into SAH. Sites where key chemical changes occur during this catalytic reaction are circled in red. (**B**) Methyltransferases catalyze the methylation of the ε-amino group of the target lysine residue on the protein, subsequent methylation of target lysine residue results in mono-, di-, and trimethylated lysine.

Protein methylation first drew significant research attention in 1964 when its role in gene expression through the modification of histones was discovered [10]. Numerous methyltransferase (MTase) enzymes have been identified that mediate protein methylation by catalyzing the transfer of a methyl group from the universal methyl donor, S-adenosylmethionine (SAM), to various substrates, converting SAM into S-adenosylhomocysteine (SAH) [11] (Figure 1A). Generally, protein lysine methylation involves the chemical attachment of mono-, di-, or trimethyl groups (designated as Kme1, Kme2, or Kme3, respectively) to the ε-nitrogen of specific lysine residues on target proteins [12] (Figure 1B). The dynamic process of protein lysine methylation is controlled by enzymes known as lysine methyltransferases (KMTs), which add methyl groups to lysine residues, and lysine demethylases (KDMs), which remove methyl groups [13]. For a better descriptive approach, KMTs are commonly referred to as “writers,” while KDMs are called “erasers” [14,15].

Although lysine methylation was initially associated with histones, many lysine KMTs, KDMs, and reader domains have now been identified that act on or recognize histone proteins. However, lysine methylation also occurs in non-histone proteins, both nuclear and cytoplasmic, and the number of these proteins continues to grow [16]. Similar to histone lysine methylation, the primary function of non-histone lysine methylation is to regulate protein–protein interactions [17], which in turn influence protein stability, subcellular localization, and DNA binding [16]. To date, nearly 20 non-histone substrates have been discovered for KMTs and KDMs [17]. For example, LSD1 has been reported to demethylate p53 at lysine 370, where monomethylation and demethylation appear to have different functional implications [18]. Additionally, SMYD3 mediates lysine methylation of the kinases VEGFR1 and MAP3K2, which are involved in cancer cell proliferation and angiogenesis [19]. Heat shock proteins (HSPs), which are overexpressed in a wide range of human cancers, are also subject to lysine methylation, highlighting their role in tumor progression and treatment resistance [20,21]. Together, the protein methylation in human cell is realized within the regulatory system encompassing both histone and nonhistone protein modifications and serving for directed regulation of protein biological processes.

## 2. Structures and Functions of KMTs and KDMs

**Figure 2:**
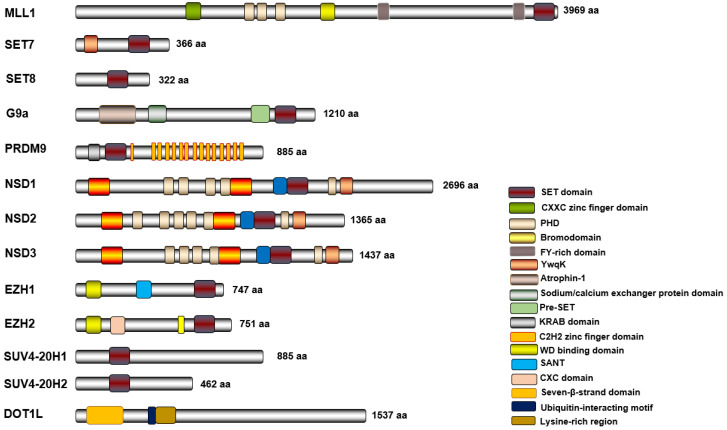
Schematic representation of the domains of selected methyltransferases. Different domains are represented in different colors.

To date, more than 50 lysine methyltransferases (KMTs) and 20 lysine demethylases (KDMs) have been reported [22]. Bioinformatics analyses predict that the human genome encompasses over 100 KMTs targeting more than 1000 human proteins [23]. KMTs exhibit a modular structure with multiple functional domains [24] (Figure 2). For example, the large histone KMT mixed-lineage leukemia protein 1 (MLL1) consists of several histone lysine methylation binding domains [13]. In contrast, smaller KMTs, such as SUV4-20H1 and DOT1L, contain only one or two lysine methylation binding domains. Most known KMTs feature a conserved SET (Su(var)3-9, Enhancer of Zeste, Trithorax) domain responsible for enzymatic activity [25]. In addition to the SET domain, many KMTs possess other defined protein domains or homologous sequences that help classify them into distinct subfamilies [26].

The biochemistry, structural, and molecular biology of methyltransferases (MTases) related to human biology, epigenetics, and disease have been extensively covered in previous reports [27,28]. Currently, five main classes (I–V) of S-adenosylmethionine (SAM)-dependent MTases are recognized, categorized based on their structural features [29]. Class I includes more than 33 family members, most of which contain a seven-stranded β-sheet flanked by α-helices. Class II features the reactivation domain of methionine synthase, which contains long β-strands and binds to AdoMet in a shallow groove on the domain’s surface. Class III possesses an AdoMet-binding site situated between two α/β domains, with a groove in the N-terminal domain proposed as the active-site cleft. Class IV represents the SPOUT family of RNA MTases, which features a novel knot structure at the C-terminus contributing to the AdoHcy-binding site. Class V consists of the SET domain–containing histone-lysine N-MTase family, formed by three small β-sheets [30]. The methylation catalyzed by these enzymes plays a crucial role in biosynthesis, signal transduction, protein repair, and various physiological processes. Based on sequence and structural homology, KMTs can also be divided into two families according to their catalytic domains. The first family includes the suppressor of variegation 3–9 (Su(var) 3–9), Enhancer of Zeste (E(z)), and Trithorax (SET) family, which contains the unique functional SET domain originally identified in Drosophila polycomb proteins. The second family comprises the disrupter of telomeric silencing 1-like (DOT1L) family [31,32,33], together accounting for approximately 90% of all human methyltransferases.

**Figure 3:**
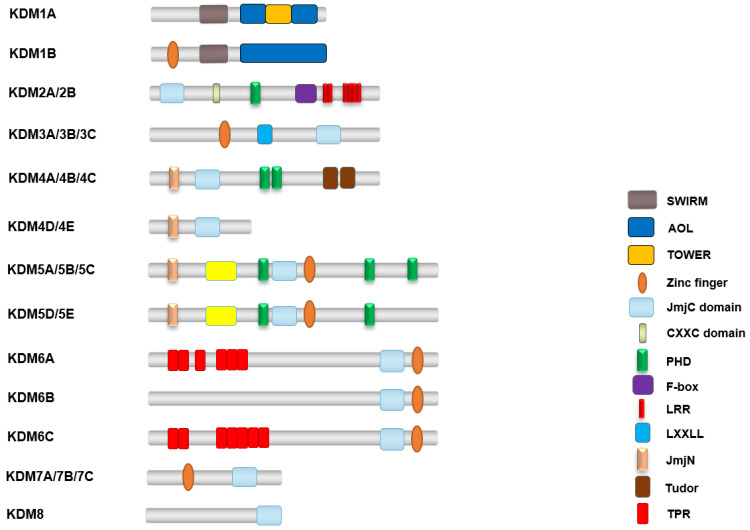
Schematic representation of the domains of demethylases. Different domains are represented in different colors.

There are two major groups of demethylases based on their enzymatic activity as follows: FAD-dependent amine oxidases and Fe (II) and α-ketoglutarate-dependent hydroxylases [33]. These groups are further classified into two classes and eight subfamilies based on their catalytic mechanisms and sequence homology (Figure 3). The first class includes the KDM1 subfamily, while the second class comprises the KDM2-8 subfamilies, which are characterized by the presence of catalytic JmjC domains and are dependent on Fe (II) and 2-oxoglutarate (2-OG) [34].

**Figure 4:**
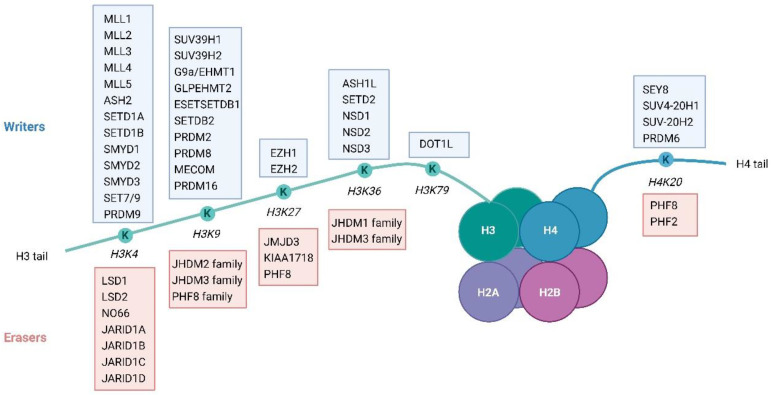
Schematic depiction of a nucleosome showing principal lysine methylation sites on histones H3 and H4. The figure presents the known writers (methyltransferases) and erasers (demethylases) associated with each lysine methylation site.

KDMs function by hydroxylating the methyl groups at the location of interest, followed by the formation of formaldehyde as a by-product [35,36]. Additionally, known mammalian demethylases have been categorized into multiple families (KDM1, KDM2, KDM3, KDM4, KDM5, KDM6, KDM7, KDM8, JARID2, and JmjC families) based on their unique domains [36,37,38]. In humans, the canonical lysine methylation sites are found on histone H3 at lysine 4 (H3K4), lysine 9 (H3K9), lysine 27 (H3K27), lysine 36 (H3K36), and lysine 79 (H3K79), and on histone H4 at lysine 20 (H4K20) [9,21,25,33,39] (Figure 4). These modifications regulate an array of chromatin functions. In addition to these canonical sites, there are several less well-characterized sites of lysine methylation on core histones, such as H3K23me, H3K63me3, H4K5me1, and H4K12me1 [40,41,42,43].

Together, the substantial numbers of lysine methylation sites and differentially methylated states present in histones illustrate the potential complexity of this signaling system. Histone lysine methylation is implicated in a wide spectrum of biological processes, including cell division, cell cycle regulation, survival, proliferation, metabolism, development, immunoregulation, transcriptional regulation, and genome stability [44,45,46]. Consequently, the dysregulation of histone lysine methylation has been linked to the development and progression of multiple diseases, including cancer and genetic and metabolic disorders.

Overall, this review highlights our current knowledge on the role of lysine methylation, the importance of methyltransferases and demethylases in glucose and lipid metabolism, and provides evidence of their implications for disease etiology.

## 3. Roles of Lysine Methylation in Glucose Metabolism

**Figure 5:**
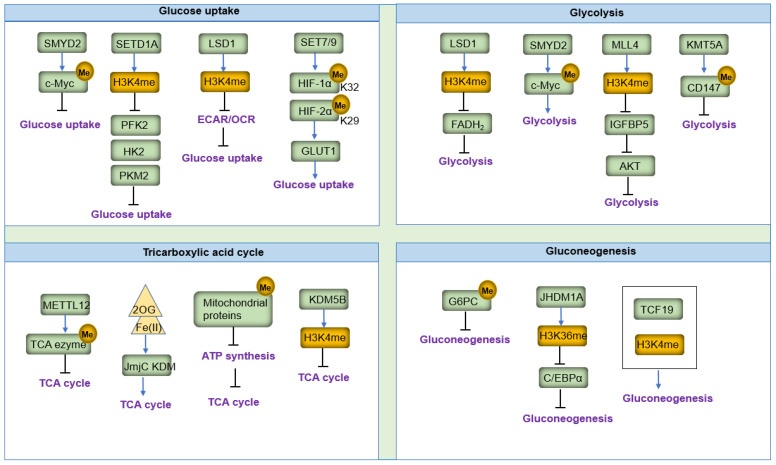
Alterations of lysine methylation and targeted genes affecting glucose metabolism. The figure highlights the involvement of lysine methyltransferases (KMTs), lysine demethylases (KDMs), and their respective histone methylation marks in processes such as glucose uptake, glycolysis, the tricarboxylic acid cycle, and gluconeogenesis.

Glucose metabolism is crucial for cell cycle regulation, growth, apoptosis, and energy metabolism [47]. The central processes of glucose metabolism are glycolysis and gluconeogenesis [48]. Disruptions in glucose metabolism often result from energy and substance metabolism imbalances [49]. Understanding the regulation and molecular mechanisms of glucose metabolism is essential for comprehending the basis of many metabolic disorders. Recent studies have revealed that lysine methylation tightly regulates rate-limiting enzymes in glucose and lipid metabolism [50,51]. Here, we summarize our current knowledge of lysine methylation in glucose and lipid metabolism (Figure 5). We outline the basic regulation of glucose and lipid metabolism, highlighting their connections with lysine methylation in both normal physiology and disease, using multiple well-documented examples.

### 3.1. Glucose Uptake

Glucose metabolism is a primary cellular process that balances energy in response to various regulatory factors [52]. Glucose homeostasis is maintained by a balance between hepatic glucose production and glucose uptake, making glucose uptake a critical determinant in cellular glucose metabolism [39]. Numerous studies suggest that lysine methylation affects glucose uptake efficiency in carbohydrate metabolism [53]. For instance, the methyltransferase SMYD2 is reported as a regulator of glucose uptake. Inhibition of SMYD2 alters glucose uptake by methylating c-Myc and increasing its protein stability [54]. SETD1A, an H3K4 lysine methyltransferase, reduces glucose uptake by decreasing the expression of genes such as Hexokinase 2 (*HK*2), 6-phosphofructo-2-kinase (*PFK*2), and pyruvate kinase M2 (*PKM*2) [55]. The SET domain–containing lysine methyltransferase Set7 methylates HIF-1α at lysine 32 and HIF-2α at lysine 29, inhibiting the expression of HIF-1α/2α targets. Knockdown or inhibition of Set7 under hypoxic conditions increases glucose uptake, demonstrating that Set7-mediated lysine methylation negatively regulates HIF-1α transcriptional activity and HIF-1α-mediated glucose homeostasis [56]. Lysine-specific demethylase-1 (LSD1, KDM1A) plays an essential role in maintaining metabolic balance [57], and inhibition of LSD1 causes glucose uptake decreasing and inducing metabolic imbalance [57]. LSD1 knockdown significantly suppresses the invasive activity and glucose uptake of cancer cells, reduces their extracellular acidification rate (ECAR), and increases their oxygen consumption rate (OCR) and OCR/ECAR ratio, thus activating the glycolytic pathway and mitochondrial respiration in esophageal cancer cells [58].

The limiting step in glucose metabolism is glucose transport through the cell membrane via glucose transport proteins [59]. There are two families of cellular glucose transporters: GLUT and sodium-dependent glucose transporters (SGLTs) [60]. Studies have shown that lysine methylation regulates these critical transporters in glucose transport. Lysine methylation directly influences GLUT activity. For example, methyltransferase SET7/9 reduces HIF-1α methylation at lysine 32, increasing HIF-1α levels and recruitment of HIF-1α target genes GLUT1, promoting glucose transport and angiogenesis [61]. Additionally, post-translational modifications (PTMs) indirectly affect GLUT levels. For example, modifications of chromatin structure, including dimethylated histone H3K4, were significantly increased at the GLUT4 promoter region in female pup muscle following a maternal low-protein (LP) diet [62]. GLUT4 is encoded by the Slc2a4 gene. Studies show that increased H3K9me3 in the Slc2a4 promoter enhancer segment reduces GLUT4 expression in skeletal muscle and worsens glycemic control in diabetes, pointing to H3K9me3 of the Slc2a4 promoter as a potential target for treating diabetes [63]. The expression of GLUT1 can be regulated by lncRNA HOTAIR. Silencing of lncRNA HOTAIR, induced by remodeling chromatin H3K4 trimethylation, reduces the recruitment of NF-κB on the GLUT1 (SLC2A1) promoter region [64], thereby suppressing GLUT1 expression in lauric acid (LA)–mediated metabolic reprogramming.

### 3.2. Glycolysis

Glycolysis, the first step in the breakdown of glucose, produces high-energy molecules ATP and NADH by converting glucose into pyruvate [65]. Numerous studies have shown that KMTs and KDMs regulate glycolytic processes by affecting the translocation, content, and stability of rate-limiting enzymes. For example, the Jumonji C (JmjC) class of KDMs facilitates the removal of post-translational methylation from modified lysine residues and is currently being studied as oxygen signaling proteins [66]. These KDMs use available molecular oxygen (O_2_), Fe(II), and α-ketoglutarate (αKG) to catalyze the hydroxylation of substrates and initiate the demethylation of their substrates. FAD, a crucial coenzyme involved in glycolysis, is reduced to FADH_2_ catalyzed by LSD1/KDM1 during the oxidation of the methylated lysine substrate, generating an imine intermediate [67]. FADH_2_ can also be reoxidized to FAD, and this FAD/FADH_2_ redox ratio regulated by KDMs is an important rate-limiting factor for glycolysis [68].

Reprogrammed energy metabolism, particularly aerobic glycolysis (the Warburg effect), has emerged as a hallmark of cancer [69]. The protein lysine methyltransferase SMYD2 functions as an oncogene and is implicated in various malignant phenotypes of human cancers [54,70,71]. Bioinformatic analysis revealed a novel link between SMYD2 expression and aerobic glycolysis [54]. SMYD2 methylates c-Myc, increasing its protein stability, and promoting hepatocellular carcinoma progression by reprogramming glycolysis [54]. SMYD2 also alters the methylation status of p53 and inhibits its transcriptional activity; SMYD2 knockdown induces a metabolic shift from aerobic glycolysis to oxidative phosphorylation, while SMYD2 overexpression promotes glycolytic metabolism in cervical cancer cells [72]. MLL4, an H3K4 methyltransferase, acts as a potent tumor suppressor in melanoma. Loss of MLL4 leads to enhancer reprogramming on the tumor suppressor IGFBP5, resulting in activation of AKT and rewiring of glycolytic pathways [73]. Additionally, lung-specific loss of MLL4 promotes lung tumorigenesis and upregulates pro-tumorigenic glycolysis programs in lung cancer [74]. H3K27 is also important in tumor glycolysis. Glucose-derived α-KG maintains low H3K27me3 levels, and inhibition of key enzymes in glycolysis increases H3K27me3, prolonging survival in animal models [75]. The H3K4-specific methyltransferase SETD1A plays a crucial role in gastric cancer; knockdown of SETD1A reduces lactate production and suppresses glycolysis by decreasing the expression of several glycolytic genes [55]. Knockdown of SETD1A decreases H3K4 methylation on the HK2 and PFK2 promoters and reduces HIF1α recruitment, necessary for promoting the transcription of glycolytic genes. The glycoprotein CD147 is dimethylated to CD147-K234me2 by lysine methyltransferase 5A (KMT5A); overexpression of CD147-K234me2 and KMT5A enhances glycolysis and lactate export in non-small cell lung cancer (NSCLC) cells [76]. The crosstalk between glycolysis and mitochondrial metabolism is a vital mechanism for cell energy metabolism [77]. Lysine demethylase 5A (KDM5A) promotes pancreatic cancer progression by redirecting mitochondrial pyruvate metabolism [78]. Another demethylase, LSD1, plays an essential role in maintaining glycolytic metabolism activity [79]. Inhibition of LSD1 is accompanied by the activation of mitochondrial respiration, driven by the activation of a set of mitochondrial metabolism genes with a concomitant increase in methylated histone H3K4 in the promoter regions. KDM5A and LSD1 also highlight the importance of KDMs in glycolysis. The activity of KMTs and KDMs is regulated by microRNAs. For example, methyltransferase EZH2 promotes tumor glycolysis, while miR-138 inhibits EZH2 by decreasing H3K27 methylation [80]. Moreover, miR-448 downregulates the expression of demethylase KDM2B, leading to elevated methylation levels and the activation of glycolytic metabolism in gastric cancer.

### 3.3. Tricarboxylic Acid Cycle

The tricarboxylic acid (TCA) cycle is the major final common pathway for the oxidation of carbohydrates, producing large amounts of ATP via oxidative phosphorylation [81,82]. Many enzymes participate in delivering reducing equivalents to the electron transport chain during the oxidative phosphorylation process [83], a large percentage of which are regulated by lysine methylation. For example, methyltransferase-like protein 12 (METTL12), a member of the 7β-strand methyltransferase family, methylates lys-368 of citrate synthase in an external surface region close to its catalytic site [84]. Citrate synthase is a critical enzyme of the TCA cycle in mitochondria. Trimethylation of a lysine residue in mitochondrial apocytochrome c by Ctm1p has been observed in *Saccharomyces cerevisiae* [85]. Four other major mitochondrial proteins have been reported to contain trimethylated lysines, including citrate synthase [86], the β-subunit of the electron transfer flavoprotein (ETFβ) [87], the ADP/ATP translocase [88], and the c-subunit in the rotor of the ATP synthase [89]. All these proteins occupy important positions in the TCA cycle. Additionally, lysine methylation by the mitochondrial methyltransferase FAM173B optimizes the function of mitochondrial ATP synthase [90]. The Set7 lysine methyltransferase regulates plasticity in oxidative phosphorylation necessary for trained immunity induced by β-glucan [91]. Demethylases also play vital roles in the TCA cycle. Studies show that the JmjC KDMs are Fe(II) and 2-oxoglutarate (2OG)-dependent oxygenases, some of which are involved in the TCA cycle [92,93]. Altered levels of TCA cycle intermediates and the associated metabolites D- and L-2-hydroxyglutarate (2HG) can cause changes in chromatin methylation status [94]. Overexpressing KDM5B in response to dosing with TCA cycle metabolite pro-drug esters regulates H3K4 methylation status, suggesting the potential for KDM5B inhibition by TCA cycle intermediates [95].

### 3.4. Gluconeogenesis

Gluconeogenesis is a crucial pathway in glucose metabolism, playing a vital role in maintaining blood glucose levels [96]. Dysregulation of gluconeogenesis is a key pathological feature of various diseases, including type 2 diabetes, obesity, and tumors [97]. The regulation of gluconeogenesis involves the following two primary mechanisms: direct regulation through rate-limiting enzymes and indirect regulation through nonrate-limiting enzymes [4]. The activity of these enzymes is extensively regulated by lysine methylation [98]. For instance, Glucose-6-phosphatase (G6PC) is critical for glucose homeostasis as it catalyzes the final steps of gluconeogenesis [99]. The binding of glucocorticoid receptor to the G6PC promoter is accompanied by hypomethylation of the promoter, and the levels of H3K9me3 and H3K4me3 on the G6PC promoter influence hepatic activation of G6PC gene expression, which can contribute to hyperglycemia [100]. The H3K36 demethylase JHDM1A regulates gluconeogenesis through its demethylation activity. Mechanistically, JHDM1A regulates the expression of a major gluconeogenic regulator, C/EBPα, by demethylating dimethylated H3K36 on the C/EBPα locus [101]. In vivo, silencing JHDM1A promotes liver glucose synthesis, while its exogenous expression reduces blood glucose levels. Transcription factor 19 (a) interacts with H3K4me3 and controls gluconeogenesis via the nucleosome-remodeling-deacetylase complex [102], highlighting the transcriptional regulation of gluconeogenesis and the roles of lysine methylation in maintaining metabolic homeostasis.

## 4. Roles of Lysine Methylation in Lipid Metabolism

Lipid metabolic reprogramming is a hallmark of cell metabolism in various diseases. Aberrant lipid metabolism has thus emerged as a potential metabolic vulnerability in these conditions. In lipid metabolism, numerous metabolites can regulate gene expression and activate various pathways. Additionally, increasing evidence has shown that lipid metabolism can lead to the transient generation or accumulation of toxic lipids, resulting in endoplasmic reticulum (ER) stress, which in turn regulates post-translational modifications (PTMs) of immune checkpoints. Understanding the factors that regulate lipid metabolism may provide new potential therapeutic strategies.

**Figure 6:**
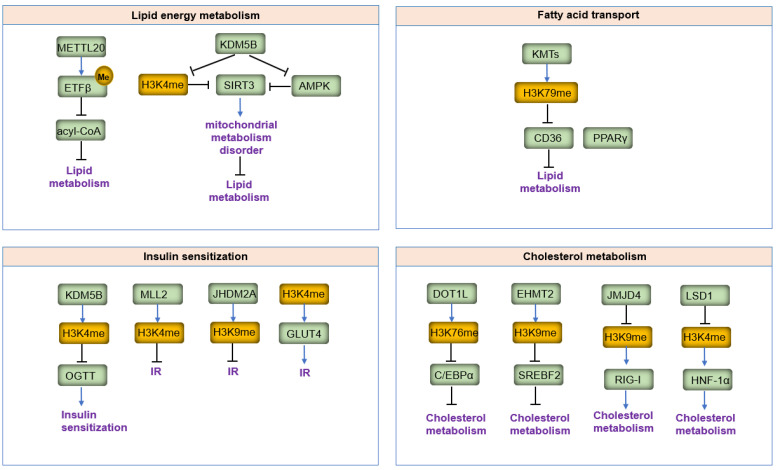
Alterations of lysine methylation and targeted genes affecting lipid metabolism. The figure depicts the involvement of lysine methyltransferases (KMTs), lysine demethylases (KDMs), and their respective histone methylation marks in processes such as lipid energy metabolism, fatty acid transport, insulin sensitization, and cholesterol metabolism.

This review gathers recent findings on the role of lysine methylation in lipid metabolism (Figure 6), highlighting how lysine methylation influences lipid metabolism by affecting key proteins at critical steps.

### 4.1. Lipid Energy Metabolism

Mitochondria are the primary intracellular organelles involved in energy production, cell metabolism, and cell signaling [103]. The involvement of mitochondria in lipid energy metabolism is a significant factor in tumor development and metastasis [104]. In mammalian mitochondria, although protein lysine methylation is not the most common post-transcriptional modification, it displays vital biological function. METTL20 is the first lysine methyltransferase to be found to be associated with mitochondria [87,105,106]. It methylates the β-subunit of the mitochondrially localized electron transfer flavoprotein (ETFβ) at Lys-200 and Lys-203, reducing its ability to receive electrons from medium-chain acyl-CoA dehydrogenase and glutaryl-CoA dehydrogenase. Additionally, Lysine demethylase 5B (KDM5B) has been found to be associated with mitochondria. KDM5B binds to the SIRT3 promoter and its overexpression triggers mitochondrial metabolism disorders and oxidative stress by directly inhibiting SIRT3 expression through demethylating H3K4me3 or indirectly repressing the AMPK pathway-regulated SIRT3 expression. This downregulation of SIRT3-mediated mitochondrial lipid metabolism is significant in diabetic neuropathy [107].

### 4.2. Fatty Acid Transport

In mammals, white adipocytes are specialized cells for the storage of energy (in the form of triacylglycerols) and for energy mobilization (as fatty acids) [108]. The metabolism of white adipocytes plays an essential role in whole-body homeostasis [109]. During lipid metabolism and energy production, triglycerides stored in adipose tissue are mobilized and decomposed into free fatty acids and glycerol, which are then released into the blood and transported to tissues requiring energy. This process involves various transporters, such as fatty acid translocase (FAT), also known as cluster of differentiation 36 (CD36) [110]. The interaction between CD36, lipid dysmetabolism, and obesity has been identified in various models and human studies [111]. CD36 facilitates the transport and uptake of long-chain fatty acids, and lysine methylation plays a key regulatory role in CD36-mediated fatty acid transport [111]. CD36 exhibits high levels of H3K79me1 methylation centered 41 kb upstream of the transcription start site. The intergenic enrichment of H3K79 monomethylation upstream of the CD36 gene correlates with PPARγ occupancy [112]. Importantly, intergenic monomethylation of H3K79 and H3K4 is significantly increased in adipocytes relative to preadipocytes, corresponding with CD36 transcription.

### 4.3. Insulin Sensitization

Most human cells utilize glucose as the primary energy substrate, and cellular uptake of glucose requires insulin [113]. Reduced insulin sensitivity commonly leads to insulin resistance [114]. The causes of insulin resistance in obesity and type 2 diabetes mellitus (T2DM) are not limited to impaired insulin signaling but also involve the complex interplay of multiple metabolic pathways [115]. Lipid metabolism disorders are closely connected with insulin resistance [116]. Lysine methylation of proteins by metabolites and lipids can alter protein function, contributing to insulin resistance. Sequencing data analysis revealed 2644 regions differentially enriched in H3K4me3 in first-degree relatives of type 2 diabetics compared to controls, with significant enrichment in mitochondrial-related genes [117]. The significant reduction in H3K4me3 abundance on these genes contributes to early insulin resistance in type 2 diabetics.

H3K4 demethylase KDM5B knockout decreases insulin secretion by improving insulin sensitivity, thus maintaining normoglycemia following an oral glucose tolerance test (OGTT) [118]. This aligns with studies showing that H3K4 methyltransferase Set7/9 deletion leads to glucose intolerance [119], while MLL2 deficiency impairs glucose tolerance and induces insulin resistance [120]. Studies have also shown that mutations in MLL2 lead to impaired glucose tolerance and insulin resistance, as well as impaired insulin secretion in isolated islets [120], These findings reveal that gene expression controlled through histone lysine methylation is a significant mechanism involved in insulin sensitization.

JHDM2A (JmjC domain–containing histone demethylase 2A, also known as JMJD1A) catalyzes the removal of H3K9 mono- and dimethylation through iron and α-ketoglutarate-dependent oxidative reactions [121]. Modulation of H3K9 mediated by JHDM2A is associated with obesity and insulin resistance in obesity and metabolic syndrome [111]. GLUT4 is an insulin-regulated glucose transporter involved in insulin sensitivity [122]. Dimethylated histone H3K4 is detected at significantly increased levels at the GLUT4 promoter region in female pup muscle following a maternal low-protein diet, which leads to increased insulin resistance [62]. Lysine methylation affects insulin sensitization not only in mammals but also in *C. elegans*. Mutations in a class of putative H3K9 mono/dimethyltransferase genes (met-2, set-6, set-19, set-20, set-21, set-32, and set-33) induce synergistic lifespan extension in the long-lived DAF-2 (insulin growth factor 1 [IGF-1] receptor), resulting in a reduction of the insulin signaling pathway [123].

### 4.4. Cholesterol Metabolism

Cholesterol is an essential component of cellular membranes, playing a key role in regulating membrane structure and fluidity [124]. Furthermore, cholesterol serves as a precursor for steroid hormones, oxysterols, and bile acids, all of which are essential for maintaining many of the body’s metabolic processes [125]. Post-translational modifications (PTMs) regulate the key enzymes and proteins involved in cholesterol metabolism [126], including lysine methylation.

DOT1L mediates the methylation of histone H3K79. Conditional knockout of Dot1l in mouse cerebellar granule cells leads to significant transcriptional changes in genes involved in cholesterol and lipid metabolism [127]. A low-protein diet causes decreased histone H3K9me1 and H3K27me3 levels on the HMGCR and CYP7α1 genes, implicating potential long-term consequences in cholesterol homeostasis later in adult life [128]. Euchromatic histone-lysine N-methyltransferase 2 (EHMT2) is a histone methyltransferase that catalyzes H3K9me1 and H3K9me2. EHMT2 inhibition lowers H3K9me1 and H3K9me2 levels at the promoter of SREBF2, a master regulator of cholesterol biosynthesis [129]. This results in induced SREBF2 expression and altered cholesterol metabolism-dependent autophagy.

The H3K9 methyltransferase SETDB1, found in complex with Kap1, regulates the expression of genes associated with cholesterol secretion and triglyceride synthesis [130]. KDMs also exhibit important biological functions in cholesterol metabolism. For example, retinoic acid-inducible gene-I (RIG-I) is low-expressed in HFD, enhancing cholesterol synthesis and steatosis. However, JMJD4-demethylated RIG-I prevents these processes [131]. Another example is the lipid-associated single nucleotide polymorphism gene region (LASER), which binds to lysine demethylase LSD1. LASER knockdown enhances LSD1 targeting to genomic loci, resulting in decreased H3K4me1 levels at the promoter regions of the HNF-1α gene. Conversely, LSD1 knockdown abolishes the effect of LASER on HNF-1α and PCSK9 expressions, dramatically reducing intracellular cholesterol levels and affecting the expression of genes involved in cholesterol metabolism [132].

## 5. Therapeutic Implications of KMT and KDM Inhibitors in Diseases Associated with Dysregulated Glucose and Lipid Metabolism

Post-translational modifications of histones by lysine methyltransferases (KMTs) and lysine demethylases (KDMs) play crucial roles in regulating gene expression and transcription. These modifications are implicated in many diseases [133]. Many of these enzymes also target various non-histone proteins, impacting numerous essential biological pathways. Given their significant biological functions and implications in human diseases, there has been growing interest in assessing these enzymes as potential therapeutic targets [134]. Consequently, the discovery and development of inhibitors for these enzymes have become a highly active and fast-growing research area over the past decade. In this review, we discuss the discovery, characterization, and biological applications of KMT and KDM inhibitors, with an emphasis on their implications in diseases related to glucose and lipid metabolism. We also explore the challenges, opportunities, and future directions in this exciting field of research.

### 5.1. Diabetes

Diabetes mellitus is a global public health challenge with high morbidity. Type 2 diabetes mellitus (T2DM) accounts for 90% of diabetes cases worldwide [135]. T2DM is characterized by absolute or relative insufficiency of insulin secretion and decreased sensitivity of target organs to insulin, leading to metabolic disorders involving fat, protein, water, electrolytes, and other substrates [136]. Despite glucose-lowering treatments, T2DM often progresses, with 50% of individuals requiring insulin therapy within 10 years [137]. Although the etiology and pathogenesis of T2DM remain unclear, its occurrence is related to insufficient insulin secretion or insulin resistance (IR), which is often closely associated with glucose and lipid metabolism [138,139]. Proteins and enzymes involved in lysine methylation are important targets for T2DM therapy due to their role as regulators of IR.

For example, increases in H3K4me1 and H3K9me2 at the promoter of the glucose transporter gene Glut2 are positively correlated with the progression of T2DM [140]. Preventing the progression of T2DM could potentially reverse these abnormal histone modification patterns. Epigenome-wide studies of histone modifications in tissues have shown that H3K4me1/2/3, H3K36me2/3, and H3K79me2 are correlated with the transcriptional activation of proteins involved in glucose and lipid metabolism [141]. These modifications play critical roles in specific promoters and enhancers of islets and in the pathogenesis of T2DM.

Based on the regulatory relationship between targeting histone methylation epigenetic marks and the expression levels of writers or erasers related to T2DM, various drugs and chemicals have been designed for T2DM treatment [141]. For example, Lactobacillus, a modulator of H3K79me2 and H3K27me3 methylation, is regarded as a potential treatment for T2DM. Lactobacillus supplementation prevents H3K79me2 methylation and H3K27me3 demethylation, thereby altering metabolic disorders in T2DM by regulating IR [142,143]. Metformin, a more commonly used drug for T2DM, directly targets the H3K27me3 demethylase KDM6A and can reverse the H3K36me mark in prediabetic and diet-induced obesity mouse models [144,145]. Specific inhibitors of KMTs and KDMs are also used as targeted drugs in T2DM treatment. For instance, GSK126, an EZH2-specific inhibitor, increases H3K27me3 levels in adipocytes, modulating lipid metabolism by promoting adipocyte differentiation in diet-induced obese mice [146]. The MLL1-specific inhibitor MI-2 reduces chronic inflammation and affects glucose metabolism in patients with diabetes, making it an ideal therapeutic drug for T2DM [147].

### 5.2. Obesity

Obesity is the most significant nutritional disorder in the developed world [148]. The roles of physical activity and diet in the etiology of obesity are well established [149]. Traditional treatments for obesity include lifestyle changes, nutritional education, modification, and increased exercise [149,150]. Given the close relationship between obesity and lipid and glucose metabolism, many therapeutic methods for obesity involve lysine methylation mechanisms.

Histone lysine demethylase 6a (KDM6A) mediates the removal of repressive trimethylation from H3K27me3 to activate target genes involved in glucose and lipid metabolism [151]. The level of H3K27me3 regulated by KDM6A affects the expression of Cryptochrome 1 (Cry1) in the hypothalamus of diet-induced obese mice [152]. Consequently, GSK-J4, a KDM6A inhibitor, serves as an attractive drug for obesity and metabolic disorders. GSK-J4 reduces Cry1 expression and sensitizes leptin signaling, thereby combating obesity-related diseases [152].

In addition to its role in diabetes, the EZH2 inhibitor GSK126 is also an important drug for obesity. GSK126 inhibits the differentiation of mouse embryonic fibroblasts (MEFs) into white adipocytes but promotes their differentiation into brown/beige adipocytes [153]. The histone demethylase Jumonji domain–containing protein-3 (JMJD3), which regulates the trimethylation of histone H3 on lysine 27 (H3K27me3), is another target for obesity treatment [154]. The JMJD3 inhibitor GSK-J4 can inhibit the differentiation of MEFs into brown/beige adipocytes [155]. Gomisin N (GN), a physiological lignan derived from *Schisandra chinensis*, shows potential as a novel agent for preventing and treating obesity [156]. GN inhibits the expression of JMJD2B, another histone demethylase in the JMJD family, thereby inhibiting adipogenesis and preventing high-fat diet-induced obesity.

### 5.3. Neurodegenerative Diseases

Neurodegenerative diseases represent a significant health challenge for aging populations, characterized by neuronal dysfunction and subsequent cell death [14]. Among the contributing factors, dysregulation of glucose and lipid metabolism, including type 2 diabetes mellitus (T2DM), has been implicated in the pathogenesis of these diseases [157,158]. Notably, emerging research indicates that histone lysine methylation, previously studied in the context of neurodevelopmental and psychiatric disorders, also plays a role in neurodegenerative conditions [159].

Alzheimer’s disease (AD) stands as the leading cause of dementia, posing a growing concern globally [160]. Mounting evidence points to abnormal cerebral glucose metabolism as a prevalent feature of AD [161]. The pathology of AD is characterized by the presence of amyloid-beta (Aβ) senile plaques and tau neurofibrillary tangles (NFTs), contributing to neuronal dysfunction and demise [162]. Tau methylation, a critical post-translational modification (PTM), influences the function and stability of tau protein, with studies revealing monomethylation at specific lysine residues in AD brains [163,164]. Recent investigations underscore the involvement of lysine methyltransferases (KMTs) and lysine demethylases (KDMs) in AD pathology. For example, SETD7-mediated tau monomethylation at specific lysine residues has been linked to nuclear tau localization [165]. Moreover, KDM1A, a member of the histone lysine-specific demethylase family, has been implicated in glucose and lipid metabolism and may contribute to tau-mediated neurodegeneration [166,167]. Dysregulation of KDM1A has been associated with impaired memory function and synaptic plasticity, highlighting its potential as a therapeutic target for AD [168,169].

Parkinson’s disease (PD), the second most prevalent neurodegenerative disorder, also exhibits links to glucose and lipid metabolism [170]. Modulation of these metabolic pathways holds promise for therapeutic interventions in PD [171]. Additionally, aberrant histone demethylation has been implicated in PD pathogenesis, affecting gene expression patterns [172]. Notably, histone demethylase inhibitor GSK-J4 shows potential in rescuing dopaminergic neuron loss and motor deficits in PD animal models [173,174].

Huntington’s disease (HD) represents another neurodegenerative disorder associated with dysregulated glucose and lipid metabolism [175]. Transcriptional dysregulation, coupled with abnormal histone methylation patterns, contributes to HD pathology [14]. Furthermore, KDM5C and KDM6A have emerged as regulators of HD development, suggesting that histone demethylase inhibition could hold therapeutic promise [176].

### 5.4. Cancers

Cancer research has increasingly focused on the metabolic reprogramming that characterizes malignancy [177]. However, the metabolic heterogeneity observed among human tumors presents challenges in developing effective therapeutic strategies [178]. Metabolic rewiring, closely intertwined with epigenetic remodeling, stands as a well-known hallmark of cancer. This rewiring encompasses various metabolic pathways such as the Warburg effect, fatty acid metabolism, and heightened oxidative phosphorylation, all of which contribute to the energy demands of cancer cell growth [177]. Recent studies highlight the crucial roles played by metabolic alterations and epigenetic modifications in tumor progression [166]. One significant avenue of investigation revolves around histone lysine methylation, a key mechanism of posttranslational modifications that regulates physiological and pathological processes in cancer cells [179]. Small molecule inhibitors targeting lysine methyltransferases (KMTs) and lysine demethylases (KDMs) have been developed, showing promising selective efficacy in killing cancer cells. This suggests that targeting histone lysine methylation within glucose and lipid metabolism pathways represents a viable therapeutic strategy for cancer treatment.

DOT1L, the primary methyltransferase for H3K79, governs the expression of various genes implicated in cancer initiation and progression through its catalysis of H3K79 methylation [180]. EPZ004777, the first published molecule targeting DOT1L, has demonstrated high selectivity and resulted in a dose-dependent reduction in global H3K79 methylation [181]. Additionally, compounds like EPZ-5676 (pinometostat) have shown efficacy in inhibiting DOT1L, influencing metabolism pathways in animal models [182,183]. LSD1, another crucial player in cancer cellular processes, regulates energy metabolism and represents a potential target for cancer therapy through metabolic pathways [184]. LSD1 regulates cell energy metabolism via mitochondrial biogenesis, glucose, and lipid metabolism [57], making it a potential target for cancer treatment through metabolism pathways. EHMT1 and EHMT2, homologous SET domain–containing KMTs, have emerged as regulators of cancer metabolism, promoting glycolysis and sustaining serine-glycine biosynthesis in cancer cells [185]. EHMTs are regulators of cancer metabolism, promoting glycolysis in breast cancer cells and maintaining the activity of the serine-glycine biosynthetic pathway, which is required for cancer cell proliferation [186]. Inhibitors targeting these enzymes, such as BIX-01294 and UNC0638, have shown promise in suppressing cancer cell growth by inducing metabolic dysfunction [187,188]. In H3K27M diffuse intrinsic pontine gliomas (DIPGs), characterized by H3.3K27M mutations and reduced H3K27me3 levels, inhibiting enzymes involved in α-ketoglutarate (α-KG) production has demonstrated antitumor activity in mouse models [75]. Moreover, modulating metabolites like succinate and glutamate, as well as inhibiting the H3K27 demethylase KDM6A/6B, holds potential in altering cell proliferation rates in DIPG models.

## 6. Conclusions

In recent years, a growing body of research has highlighted the significance of lysine methylation, methyltransferases, demethylases, and methyllysine-binding proteins in various diseases. Consequently, numerous histone methylation–related proteins are under investigation as potential therapeutic targets. Glucose and lipid metabolism are fundamental biological processes essential for tissue physiology. As a crucial post-translational modification, lysine methylation plays pivotal roles in the regulation of glucose and lipid metabolism. This review provides an overview of global advancements in lysine methylation within human cells, emphasizing the contributions of lysine methyltransferases and demethylases to glucose and lipid metabolism. Notably, several lysine methyltransferases and demethylases have emerged as promising therapeutic targets over the past decade. We discuss the chemical biology and pharmacology of inhibitors targeting these enzymes, as well as targeted protein degraders, with a focus on their clinical implications in diseases such as diabetes, obesity, neurodegenerative diseases, and cancer. Targeting lysine methylation in glucose and lipid metabolism appears to be a promising therapeutic strategy for these conditions.

Despite significant progress in understanding lysine methylation and its implications for glucose and lipid metabolism in human diseases, many aspects remain to be explored. Rare lysine methylation sites, such as H3K23me, H3K63me3, H4K5me1, and H4K12me1, have received little attention and warrant further investigation. Additionally, while tau methylation plays a crucial role in Alzheimer’s disease, the lysine methyltransferases involved in tau protein methylation remain poorly understood, limiting the potential application of lysine methylation markers as therapeutic targets in glucose and lipid metabolism diseases. Furthermore, most inhibitor drugs targeting lysine methyltransferases and demethylases have been developed based on their gene expression inhibitory activity rather than their effects on glucose and lipid metabolism pathways. Therefore, the therapeutic potential of lysine methylation in glucose and lipid metabolism diseases requires more attention in future studies, particularly regarding the implications of lysine methyltransferases and demethylases for clinical therapies. Addressing these challenges will be crucial for advancing our understanding and treatment of glucose and lipid metabolism-related diseases.
